# Exploring Social Desirability Bias in Perceptions of Dog Adoption: All’s Well that Ends Well? Or Does the Method of Adoption Matter?

**DOI:** 10.3390/ani8090154

**Published:** 2018-09-13

**Authors:** Courtney Bir, Nicole Olynk Widmar, Candace Croney

**Affiliations:** 1 Department of Agricultural Economics, Purdue University, West Lafayette, IN 47907, USA; nwidmar@purdue.edu; 2 Department of Comparative Pathobiology and Animal Science, Purdue University, West Lafayette, IN 47907, USA; ccroney@purdue.edu

**Keywords:** best-worst scaling, dog acquisition, dog adoption, social desirability bias

## Abstract

**Simple Summary:**

Dog adoption is often cited as an ethical and popular method of acquisition but interpretation of the term ‘adoption’ may vary. In a nationally representative survey of the U.S., 767 respondents were asked questions regarding their opinions of dog acquisition including adoption. Within the sample, 45% had a dog; of those, 40% had adopted a dog and 47% visited a veterinarian once a year. Respondents’ preferences for the most ethical method of dog adoption were elicited using a survey instrument. Our results indicate that respondents had the largest preference share for adoption from a municipal animal shelter’ (56%) and the smallest preference share for adoption from a pet store (3%). Dog acquisition was further evaluated by creating an index of social desirability bias comparing how important respondents believed certain dog characteristics were and how important respondents believed others would rate/rank the same dog characteristics. The highest incidences of social desirability bias occurred for the dog characteristics of appearance and breed.

**Abstract:**

Dogs are a popular companion animal in the United States; however, dog acquisition is often a contentious subject. Adoption is often cited as an ethical and popular method of acquisition but interpretation of the term ‘adoption’ may vary. In a nationally representative survey of the U.S., 767 respondents were asked questions regarding their opinions of dog acquisition and adoption. Within the sample, 45% had a dog; of those, 40% had adopted a dog, and 47% visited a veterinarian once a year. A best-worst choice experiment, where respondents were asked to choose the most ethical and least ethical method of acquiring a dog from a statistically determined set of choices, was used to elicit respondents’ preferences for the most ethical method of dog adoption. A random parameters logit and a latent class model were used to estimate relative rankings of dog adoption methods. In the random parameters logit model, the largest preference share was for adoption from a municipal animal shelter (56%) and the smallest preference share was for adoption from a pet store (3%). Dog acquisition was further evaluated by creating an index of social desirability bias using how important respondents believed certain dog characteristics were compared to how important respondents believed others would rate/rank the same dog characteristics. The highest incidences of social desirability bias occurred for the dog characteristics of appearance and breed.

## 1. Introduction

In the United States dogs are a popular companion, with over 54 million households having a dog [1]. The rationales and methods by which households acquire dogs is a complex subject, which has implications for various pet industry sectors and sheltering organizations, as well as for socially responsible and ethical pet ownership [2]. In addition to providing human companionship, dogs may provide therapeutic benefits to people. These potential benefits include a myriad of reported physical, mental and social benefits, including stress buffering and promotion of healthy lifestyle choices [3]. However, results are often mixed regarding the relationship between dogs and human health. Although there are reported health benefits of dog ownership in adults, a study by Westgarth et al. (2017) found that 10-year-old students in the UK did not have significantly different weight or fitness status when compared to students without dogs [4,5]. Nevertheless, positive dog-human relationships have reportedly resulted in decreased doctor visits and improved sleep [6], the general reduction of stress and improved learning in children [7] and reduced stress, anxiety and depression in college students [8,9]. Contrarily, in a population level study of English residents, Ding et al. (2018), found that dog ownership was not associated with all-cause and cardiovascular disease mortality [10]. For the disabled, specially trained dogs have been shown to enhance mobility and independence, as well as improve social integration and self-perceived health [11].

Having the ability to acquire dogs, therefore, is critical to obtain not just their companionship but the purported beneficial effects of interacting with them. However, how dogs are acquired may affect the quality of human-animal interactions and may impact aspects of dog welfare. For example, the therapeutic effects of interacting with dogs appear to be at least partly influenced by who instigated the acquisition of the dog. If the idea to get a dog was the person’s own, without outside influence, then the positive effects of the human-dog relationship were stronger [11].

Sourcing of dogs is an increasingly contentious issue. Concerns range from the quality of the conditions under which dogs are reared and bred [12], to the implications of acquisition methods for dog care and welfare. Although they only sampled the Chicago area, Freiwald et al. (2014) found that how respondents acquired a dog was not an indicator of future willingness to spend money on veterinary care [13]. However, the amount of money spent on dog care, including veterinary visits, has been steadily increasing since 1960 [14]. In 2018, pet owners spent $23.05 billion on food and 15.42 billion on veterinary services [15]. This increase in spending may be related to the idea of dogs as members of the family [14], which may in turn influences views about responsible ways of acquiring them. Nonetheless, perceptions of ethical methods of dog acquisition vary amongst people.

In a survey of U.S. residents, Bir et al. (2017), used both Likert-scale questions and best-worst scaling methodology, where respondents were asked to choose the most ethical and least ethical method of acquiring a dog from a statistically determined set of choices, to examine respondents’ perceptions of statements related to dog acquisition and the perceived most/least ethical ways to acquire a dog [16]. Ways to acquire a dog included in the experimental design were adoption, purchased directly from a breeder on site, online purchase directly from a breeder, purchased from online retailer, purchased from pet store, stray, gift and other. In the random parameters logit model used, respondents had a high preference share (80%) for adoption, which indicated they believed adoption was the most ethical way to acquire a dog. All other methods of acquiring a dog had preference shares less than 7%. In order to further analyze the results, Bir et al. (2017) used the latent class model and found that out of three identified classes of U.S. resident respondents, two indicated adoption was the most ethical way to acquire a dog. Although the study by Bir et al. (2017) began to determine preferences for dog adoption, the definition of adoption or additional details surrounding the term adoption were not given. Thus, respondents used their personal definition or understanding of dog adoption, which may have varied between respondents and may have been confounded by advertisements when answering questions. Many campaigns and publicly disseminated messages reinforce the notion that adoption is the most ethical way to acquire a dog [17] and the general public may internalize these messages, influencing what they perceive as the “right” answer. The findings that adoption was perceived to be the most ethical option in Bir et al. (2017) support the hypothesis that pro-adoption views currently predominate; however, cause and effect of pro-adoption campaigns could not be ascertained given the study sought only to measure perceptions and not underlying reasons for people’s views. It is important to also note that pet stores sometimes have agreements with shelters to showcase adoptable animals and many breeders advertise animals that were bred specifically for sale as “ready for adoption”, thus respondents’ beliefs regarding what constitutes adoption may vary. Although including adoption as a way to acquire a dog without providing more context provides a starting point for understanding views of the ethics of dog (pet) acquisition, due to its popularity as an ethical acquisition method, more research is needed to better understand public perceptions of adoption specifically.

Social desirability bias (SDB) is reflected when a respondent deviates in their stated responses from their true behavior or preference in order to make themselves look better [18]. The role of SDB in responses to questions about dog acquisition may help explain some dog characteristic preferences. Pervasive messages encouraging adoption may elicit such biases, requiring further exploration of how, or to what extent, this phenomenon may help explain dog characteristic preferences. Other studies have shown that people are strongly influenced by others when considering what breed of dog to acquire. Ghirlanda et al. (2014) found that the release of movies featuring dogs often resulted in a surge in popularity of that breed. However, spikes in breed popularity were not associated with specific characteristics that would theoretically improve dog welfare aspects, such as behavior, health or longevity. Instead, these spikes were likely a result of a particular breed being considered fashionable or a fad [19].

Given the ambiguity of people’s reasons for acquiring dogs and for adoption as a preferred method of dog acquisition, the current study aimed to expand on research conducted by Bir et al. (2017). The results of the Bir et al. (2017) study found that adoption was the most preferred method of dog acquisition. In conjunction with thoughtful comments from reviewers regarding that manuscript, discussions about how the term ‘adoption’ may be interpreted were spurred. Dog breeders may use the word adoption to refer to acquiring purposely bred puppies, or they may adopt out older breeding dogs. In general, the term ‘adopt’ is used very loosely. A quick Google search can reveal article(s) about dog acquisition titled ‘Adopting from a breeder’ [20]. Dog breeders use the term ‘adopted’ for puppies that are no longer available on their websites [21] and breeder websites often use phrases such as ‘ready for adoption‘ and ‘puppies for adoption’ [22,23,24]. It is understandable that there may be some confusion surrounding the word adoption. Additionally, pet stores often have partnerships with shelters to adopt dogs from their stores.

To further evaluate acquisition methods, the word adoption was used exclusively in a choice experiment of adoption methods. Likert-scale questions regarding general dog acquisition as well as a best-worst scaling model were used to further examine preferences for and opinions on, dog acquisition and the specific acquisition method adoption. In addition to providing more information regarding how respondents viewed the ethics of different ways of acquiring dogs, the objectives of the current study were to evaluate the potential impact of SDB bias on preferred dog characteristics and to further explore dog care habits after acquiring a dog for those respondents who had a dog.

## 2. Materials and Methods 

An online survey was conducted from 3 October, 2017 through 24 November, 2017 to collect demographic information, respondents’ opinions on dog acquisition and dog care-related questions if respondents reported a dog in the household. The survey utilized in this study was approved by the Purdue University institutional review board (IRB Protocol Number 1710019761) and the respondents remained anonymous. In total, 767 respondents completed these questions. Three hundred and forty-eight respondents indicated they had a dog at the time of the study, 142 indicated they had a dog within the past five years and 44 respondents selected both options. Lightspeed, a company that hosts an opt-in online panel of respondents was used to contact respondents. Using quotas in Qualtrics, the sample was targeted to be representative of the U.S. in terms of gender, age, income, education and region of residence. Regions of residence were as dictated in the Census Bureau Regions and Divisions [25]. Addition detail regarding the specific questions is available in the survey instrument (Supplementary file 1). 

In the survey, there were six possible responses for age: 18–24, 25–34, 35–44, 45–54, 55–65 and 65 and older. These categories were condensed to age 44 and younger and older than 45 for more meaningful interpretation. Additionally, there were 5 categories for annual pre-tax income: $0–$24,999, $25,000–$49,999, $50,000–$74,999, $75,000–$99,999 and $100,000 and higher. These categories were condensed to income of $49,999 or less and greater than $50,000. There were five categories for education: did not graduate from high school; graduated from high school, did not attend college; attended college, no degree earned; attended college, associate’s or bachelor’s degree earned; and attended college, graduate or professional degree earned. The first three categories were combined to no college degree and the last three were combined as at least a college degree.

Questions were designed to determine dog acquisition preferences amongst dog owners as well as those who do not or have not had a dog. Given that even those who do not have a dog can vote on legislation related to dog care and acquisition, it was important to gather information on a variety of respondents, including non-dog owners. The survey questions were developed by experts and were pretested before data collection. All respondents were asked a series of statements regarding dog acquisition. Questions included if dogs can be bred responsibly and ethically and methods respondents would not use to obtain a dog. The respondents were allowed to select all methods they would not use to acquire a dog. A series of 12 socially contentious questions regarding dog ownership were asked using a Likert-scale ranging from 1 (agree) to 7 (disagree) regarding dog policy and acquisition topics that included: spaying and neutering, importing dogs and shelter/rescue pets.

All respondents were asked to indicate if they currently had a dog or had a dog in the past five years. If they indicated they currently had a dog or had a dog in the past five years they were asked to indicate how they had acquired the dog. Follow up questions regarding the reason for acquiring a dog in that manner and a series of dog care questions were presented to dog owners. Respondents were asked to select all that apply from a list of potential acquisition methods. Acquisition methods included: adoption (shelter or rescue), bred them myself, purchased directly from a breeder (on site where dogs are bred/kept), purchased directly from a breeder online (via the breeder’s website), purchased from online retailer, purchased from pet store, stray, gift from friend/family member and other (i.e., parking lot). Care questions such as frequency of veterinary visits, heartworm prevention use and flea and tick prevention use were also asked of those with dogs. Cross tabulations using chi squared statistical testing were used to determine if there was a relationship between dog owner demographics, method of acquisition and dog care questions. For the purpose of the cross tabulations, the dog acquisition categories purchased directly from a breeder and purchased directly from a breeder online were condensed to the category has acquired a dog from a breeder. The categories purchased directly from a breeder, purchased directly from a breeder online, purchased from an online retailer and purchased from a pet store were condensed to the category has acquired a dog through purchase.

### 2.1. Choice Experiment: Best-Worst Scaling Experiment and Modeling

All respondents participated in a best-worst experimental design to elicit consumer preferences for different methods of dog adoption. Methods included adoption from pet store, adoption from a breeder, adoption from a municipal animal shelter, adoption from a breed rescue and adoption from a privately- owned shelter. How ethical an adoption method *j* is to respondent *i* is determine by the equation:

(1) $$I_{ij}=\lambda_{j}+\;{ℇ}_{ij}$$

where *λ_j_* is the location of adoption method j on a continuum of adoption methods from most ethical to least ethical and ${ℇ}_{ij}$ is the random error [16]. The probability that respondent *i* chooses adoption method *j* as the most ethical adoption method and adoption method *k* as least ethical is the probability that the difference between *I_ij_* and *I_ik_* is greater than all other differences available across the choice sets. Assuming the error term is *i.i.d*. type I extreme value, the probability of choosing a given most ethical-least ethical combination takes the multinomial logit (MNL) form [16,26,27]:

(2) $$Prob(j=best\;\cap k=worst)=\frac{e^{\lambda_{j}-\lambda_{k}}}{\sum_{l=1}^{J}\sum_{m=1}^{J}e^{\lambda_{l}-\lambda_{m}}-J}$$

The coefficients from the logit model are not directly interpretable, so shares of preferences are calculated to facilitate interpretation [16,26,27]; shares of preferences are calculated as:

(3) $$share_{j}=\frac{e^{\lambda_{j}}}{\sum_{k=1}^{J}e^{\lambda_{k}}}$$

The multinomial logit model was estimated but due to likely heterogeneity amongst respondents, the random parameters logit model (RPL) was also estimated. For the RPL model, confidence intervals around the preference shares were estimated using the Krinsky-Robb method and the size of the preference shares were statistically compared using the overlapping confidence interval method [28]. A second model, the latent class model (LCM), where preferences are heterogeneous across classes and homogenous within classes, was estimated to further determine preferences amongst and across respondents. Using AIC/BIC criterion, a five-class model was determined the most appropriate. The latent class model classifies individuals into one of the classes (S) based on their attitudes, demographics, and preferences [16,29]. Each of the individual respondents is assigned to an unobserved latent class by estimating simultaneously the parameters for each class [16,29]. Given membership in a specific latent class (s), the conditional probability of choices is represented as:

(4) $$\left(Prob\left(j=best\;\cap \;k=worst\right)|s\right)=\frac{e^{\lambda_{js}-\lambda_{ks}}}{\sum_{l=1}^{J}\sum_{m=1}^{J}e^{\lambda_{ls}-\lambda_{ms}}-J}$$

where the *λ_js_* and *λ_ks_* parameters are class specific [16,29,30]. The classes are unobservable but the probability of membership takes the multinomial logit form

(5) $$Prob\left(s\right)=\frac{e^{\left(\theta_{s}Z_{k}\right)}}{\sum_{s=1}^{S}e^{\theta_{s}Z_{k}}}$$

where *Z_k_* is a set of hypothesized drivers of class membership and *θ_s_* is a parameter vector that characterizes the impact that the drivers have on class membership and is normalized to zero [16,29,31]. Coefficients of the LCM classes are not interpretable, so preference shares for each latent class were also calculated using Equation (3). Additionally, for the LCM, demographics were included as independent constants in the model to add additional characterization of each of the classes. The MNL, RPL and LCM were estimated using NLOGIT6.

### 2.2. Measuring Perceptions and Social Desirability Bias in Self-Reporting

In additional to general demographic questions, respondents were asked to select from a scale of 1–4 with 1 being very important and 4 being very unimportant, which dog characteristics they believed to be most important in acquiring a dog. Human inclination may be to answer in a way that deviates from the respondent’s true behavior in an effort to make themselves look better, which is often referred to as SDB [18]. Because of the possibility of SDB, respondents were also asked to choose from the same Likert-scale which characteristics they believed others thought were the most important characteristics when acquiring a dog. The means of all responses for both questions and each characteristic were calculated and statistically compared using a *t*-test. Following Widmar et al. (2016), the difference between how important a respondent viewed a characteristic to be and how important they believed others found that characteristic was calculated and an index of those values was created [32]. Depending on the specific characteristic, either a negative or positive difference between how a respondent viewed a characteristic and how important they believed others found that characteristic indicated SDB. In other words, only cases in which an individual was seen as overstating their own “goodness” were counted as SDB, whereas overstating the “goodness” of the views of others relative to one’s self was not. The relationship between SDB and respondent demographic information was analyzed using cross tabulations.

## 3. Results

The demographics of respondents closely matched those of the greater U.S. population in terms of gender, age and income (Table 1) [25]. The test of proportions was used to determine if the percentage of respondents was statistically different than the percentage as outlined in the U.S. census. A statistically lower percentage of respondents aged 18–24 (77, 10%) responded to the survey when compared to the percentage of respondents in the U.S. census (13%). A statistically lower percentage of respondents with an income of $100,000 and higher (189, 23%) responded to the survey when compared to the U.S. census (26%). A statistically lower percentage of respondents indicated they did not graduate from high school (25, 3%) when compared to the percentage of U.S. census respondents (13%). Additionally, statistically higher percentages of respondents indicated they attended college no degree earned (184, 24%) and attended college associates or bachelor’s degree earned (246, 32%) and had earned an associates or bachelor’s degree, when compared to the percentage of U.S. census respondents 21% and 27%, respectively. A statistically higher percentage of respondents indicated their region of residence was the south (306, 40%) compared to the U.S. census (21%). A lower percentage of respondents (153, 20%) indicated their region of residence was the Midwest when compared to the U.S. census (38%).

A high percentage of respondents believed that dogs could be bred responsibly (683, 89%) and ethically (629, 82%). A small percentage of respondents indicated they would not obtain a dog via adoption through a shelter or rescue organization (79, 10%) when compared to purchasing a dog in any manner (Table 2). On a scale from 1 (agree) to 7 (disagree) respondents were asked to indicate their beliefs regarding dog acquisition (Table 3). A higher percentage of respondents selected a neutral response (4) to: the only responsible way to acquire a dog is through shelter/rescue (162, 21%), dogs in pet stores come from irresponsible breeders (228, 30%), breeding of dogs for sale is socially irresponsible (203, 26%), shelter dog populations would decrease if people stopped buying purebred dogs (199, 26%) and the sale of dogs is socially irresponsible (191, 25%) when compared to agree or disagree. A higher percentage of respondents agreed (1), when compared to disagree (7) or neutral (4), with the statements, people should be able to buy purebred dogs (259, 34%) and people should have choices as to where/how to obtain dogs (245, 32%). Results were mixed with a higher percentage of respondents selecting agree or neutral in regards to the statements all dogs should be spayed/neutered (208, 27% vs. 160, 21%), there is a dog overpopulation problem in the U.S. (225, 29% vs. 169, 22%), every shelter/rescue dog is adoptable (164, 21% vs. 161, 21%), importing of dogs for sale is irresponsible (241, 31% vs. 163, 21%) and importing dogs for adoption is irresponsible (188, 25% vs. 164, 21%) when compared to the percentage who selected disagree.

Almost half the respondents currently owned a dog (348, 45%) while 142, 19%, had kept a dog in the past five years (Table 1). The most common methods of acquisition were adoption from a shelter or rescue organization (178, 40%), gift from friend/family member (134, 30%) purchased directly from a breeder on site (90, 20%) and stray (59, 13%) (Table 4). Common responses to reason for acquiring a dog in that manner were: it was the right thing to do (208, 47%) and wanted a specific breed or type of dog (133, 30%). Ninety percent of dog owners (402) indicated that they took their dog to the veterinarian at least once a year and 55% (244) of dog owners used heart worm prevention continuously. A high percentage of dog owners (261, 59%) used flea and tick prevention continuously.

For those who indicated having a dog, the care of the dog varied based on demographics (Table 5). A higher percentage of male respondents (184, 52%) used heartworm preventative when compared to the percentage of female respondents (182, 44%). A higher percentage of respondents younger than 44 took their dog to the veterinarian (199, 57%), used heartworm prevention (185, 53%) and used flea prevention (206, 59%), when compared to those 45 and older (205/49%, 205/49% and 188/45% respectively). A lower percentage of respondents with an income of $49,999 or less took their dog to the veterinarian (161, 44%), used heartworm prevention (147, 40%) and used flea prevention (169, 46%), when compared to those with a higher income (240/60%, 216/54% and 228/57%, respectively). A higher percentage of those from the South (184, 60%) took their dog to the veterinarian, used heartworm prevention (168, 55%) and used flea prevention (190, 62%) when compared to all other regions of residence. How the respondent acquired the dog was not a good indicator of future care in terms of veterinary visits, the use of heartworm preventative, or the use of flea and tick preventative. There was not a particular way of acquiring a dog that was associated with a higher percentage of respondents who regularly took their dog to the vet, used heartworm prevention, or used flea prevention. Over 85% of those using each acquisition method indicated they took their dog to the vet, used heartworm prevention and used flea and tick prevention.

### 3.1. Choice Experiment: Best-Worst Scaling Experiment and Modeling Results

**Figure 1:**
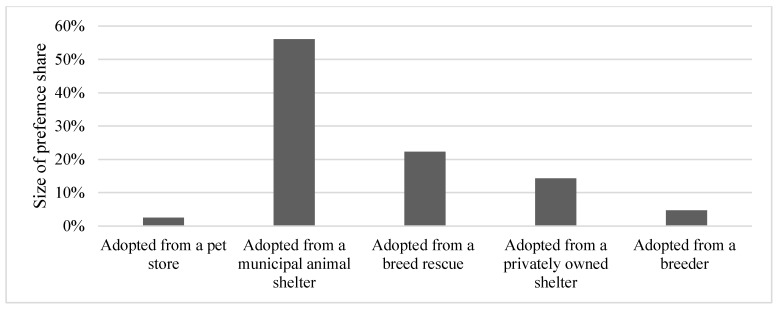
Random parameters logit model preference shares for most ethical way to adopt a dog (n = 767).

**Figure 2:**
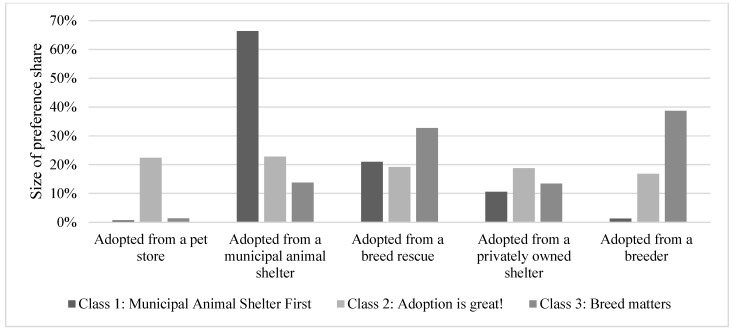
Latent class model preference shares for most ethical way to adopt a dog (n = 767).

For the random parameters logit model, all preference shares were statistically different from one another in terms of size. Respondents had the largest preference share for adopted from a municipal animal shelter (56%) (Table 6, Figure 1). The next largest preference share was for adopted from a breed rescue (22%), followed by adopted from a privately-owned shelter (14%). The two lowest preference shares were for adopted from a breeder (5%) and adopted from a pet store (3%). For the latent class model, a three-class model was found to be most appropriate (Table 6, Figure 2). The first class had a large preference share for adopted from a municipal shelter (66%) and was named “municipal shelters first”. The second class had fairly equal preference shares and was therefore named “adoption is great”. The third class had large preference shares for adopted from a breed rescue and adopted from a breeder, so this class was named “breed matters”. The collapsed demographic variables were included in the LCM model as predictors of class membership. Only the demographics, being 44 years old and younger and having at least a college degree were statistically significant. Those who were 44 years old or younger were more likely to be members of class 2, “adoption is great”, when compared to class 3, “breed matters”. Additionally, those with at least a college degree were less likely to be in class 2, “adoption is great”, when compared to class 3, “breed matters”.

### 3.2. Measuring Perceptions and Social Desirability Bias in Self-Reporting Results

All respondents were asked to indicate on a scale from 1 to 4, with 1 being very unimportant and 4 being very important, how important they found nine dog characteristics when acquiring a dog. Additionally, they were asked to indicate how important they believed others thought the same characteristics were when acquiring a dog to facilitate the calculation of SDB by characteristic. The responses were averaged and compared statistically using a *t*-test (Table 7). On average, respondents believed others thought breed was a more important characteristic (3.19) when acquiring a dog compared to how they viewed breed (2.71). Respondents also believed appearance was more important to others (3.30) than to themselves (2.81). Conversely, respondents believed compatibility with owner lifestyle was more important to them (3.38) when compared to how others viewed compatibility with owner lifestyle when acquiring a dog (3.21). Additionally, respondents believed they found behavior more important (3.40) than others did (3.32). Respondents also believed the source of the dog was more important to them (3.08) when compared to others (2.95).

**Figure 3:**
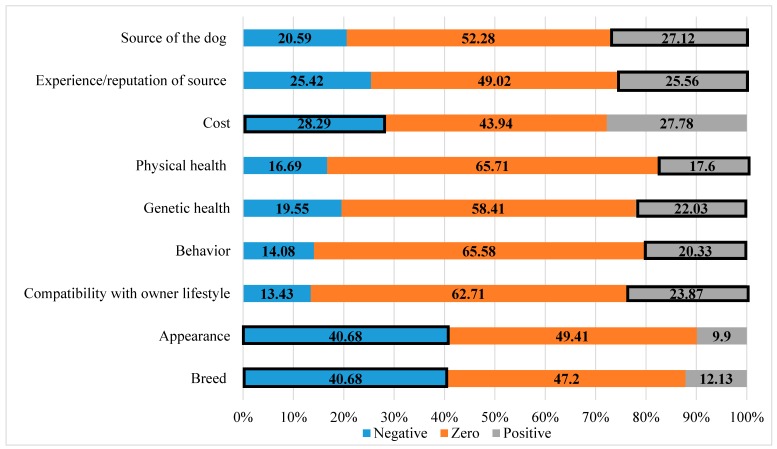
Distribution of social desirability bias for dog characteristic preferences. Social desirability bias was calculated as the difference in the score on the Likert-scale the respondent indicated for the level of importance and the score they indicated for what they thought others placed on each dog characteristic. Depending on the question, social desirability bias is indicated with having a lower score or a higher score than what others believed. Evidence of social desirability bias indicated by boxes (n = 767).

As outlined in the methods section, the score on the Likert-scale that respondents indicated for the level of importance they thought others placed on each dog characteristic was subtracted from the level of importance they selected for themselves. The distribution for that index for each of the dog characteristics is located in Figure 3. For each characteristic, SDB is indicated by either a negative or positive score in the index, based on whether finding that characteristic, unimportant or not, would be considered positive in society. For example, some respondents would be inclined, possibly due to messaging and societal pressures, to indicate that breed is not an important characteristic for them when acquiring a dog. Therefore, if the difference between their score on the Likert-scale and the level of importance they believed others placed on breed was negative (e.g., they chose a lower number on the Likert-scale for themselves and a higher number for others) then they exhibited SDB for breed. SDB was indicated by a negative score on the index for the characteristics, breed, appearance and cost; SDB was indicated by a positive score on the index for the characteristics, compatibility with owner lifestyle, behavior, genetic health, physical health, experience/reputation of source and source of the dog. A score of 0 indicated the respondent selected the same number on the Likert-scale for how important they considered a dog characteristic and how important they thought others considered a dog characteristic.

The presence of SDB for each characteristic was compared to respondent demographics using cross tabulations (Table 8). A lower percentage of males (129, 36.5%) exhibited SDB for the dog characteristic appearance, when compared to the percentage of women (182, 44.0%). For those 45 years old and older, a higher percentage (90, 21.5%) displayed SDB for physical health when compared to those 44 years of age or younger (50, 14.4%). A higher percentage of respondents with an income of over $50,000 exhibited SDB for cost when compared to the percentage of respondents with an income of $49,999 or less: 130/32.5% and 87/23.7% respectively. A lower percentage of respondents with a college degree or higher (66, 18.8%) exhibited SDB for genetic health when compared to the percentage of respondents with less than a college degree (108, 25.9%). Evidence of SDB for appearance and cost were exhibited by a higher percentage of dog owners (200/44.8%, 142/31.8%) when compared to those who did not have a dog (112/34.9%, 75/23.4%). All respondents who either indicated they currently had a dog or had a dog in the past five years were included in this category. 

## 4. Discussion

This study aimed to understand some aspects consumer preferences for dog acquisition using Likert-scales and additional questions for those who owned a dog. The study specifically focused on consumer perceptions of the acquisition method adoption by using a best-worst model. Results indicate that respondents have preferences for where the dog was adopted from and not all adoption sources were considered equal for most respondents. Additionally, this study aimed to understand the extent to which social desirability bias (SDB) might impact people’s responses to inquiries about preferred dog characteristics and to explore the care provided by those who had acquired a dog. Our findings suggest that SDB may indeed be exhibited in some of the responses received.

The results in Table 3 and Table 4 indicate that despite various publicly disseminated messages that promote dog adoption [17,33], respondents feel that dogs can be bred ethically and responsibly and that those acquiring dogs should have choices. The finding that 29% of respondents believed there was a dog overpopulation problem in the U.S. resemble those of Bir et al., 2017, where they found 29% of respondents selected agree to the same statement. This finding reflects some of the contention and disagreement by experts that there is indeed a dog overpopulation problem in the U.S., or rather a problem of too many unwanted dogs who are viewed as undesirable by prospective dog owners because of their breed, size, age, behavior or other characteristics [34]. In the U.S. an estimated 670,000 dogs are euthanized in animal shelters annually [35]. Although this number is still high, there has been a decrease in euthanasia since 1970. Rowan and Kartal (2018) propose that the decrease is likely due to an increase in adoptions and pet sterilization [14]. Twenty one percent of respondents selected agree and 26% were neutral regarding the statement shelter dog populations would decrease if people stopped breeding purebred dogs. Future research could further evaluate the reasons why people adopt dogs from shelters in particular, as well as respondent awareness of dog population levels and homelessness rates. Although 40% of respondents who currently or previously had a dog in the past five years acquired that dog through adoption, from a shelter or rescue organization, respondents still preferred having choices available. This is in agreement with economic theory that having more choices increases the probability that people’s heterogeneous preferences will be met, increasing overall utility [36].

Indicators of care including taking the dog to the veterinarian, using heartworm prevention and using flea and tick prevention varied across respondents’ demographics. Although heartworms are not contagious to people, they are preventable and pose a serious and potentially fatal threat for dogs and cats [37]. Flea and tick prevention helps improve the welfare of dogs and potentially the welfare of their human companions. Bartonella (also called cat scratch fever) is an infection that can be spread by fleas and can also be contracted by humans [38]. Given this and the fact that the vast majority of veterinarians recommend such preventatives [39], it is surprising that a low percentage of respondents reportedly used heartworm and flea and tick preventative, or only used it sometimes. It is possible that at least some respondents view heartworm and flea and tick prevention as too costly to invest in consistently, which could explain their usage only sometimes [40]. Although the average number of dogs diagnosed with heartworm disease has been increasing and no state is heartworm-free [41], it is also plausible that some dog guardians might consider the risk of heartworm disease or flea and tick infestation low for their area of residence or during certain times of year. Therefore they may be less willing to pay for continuous use of preventatives. For example, some people may not use heartworm preventative in the winter if they live in northern states, despite recommendations that continuous use is best. Alternatively, some respondents may have been confused by the choice option ‘continuous’ and unsure of the most appropriate answer if they give the medication continuously but only in the warmer months. Other studies have shown that compliance for heartworm preventative is less than 50% [39,40], which corresponds with the low use in this study. Those reporting lower incomes were less likely to take their dog to the vet and use heartworm or flea prevention, which suggests that cost may have been a factor in continuous preventive treatment. However, in the case of heartworms, treating the disease after contraction could result in a larger financial burden for the family in some areas [42], especially when weighing the possibility of the cost of treating multiple infections to the cost of preventative. Financial burdens may also be increased without regular vet visits which may catch problems earlier when they are more easily treated. Nonetheless, it is possible that for some, particularly those with lower incomes, the perceived risks to their dog’s (and potentially their) health as well as the possibility of costly treatments for parasite infestation are not sufficient to justify the immediate expense of continuous preventatives. This possibility should be explored further in future studies.

Although many pet stores partner with shelters to showcase adoptable animals, respondents in this study preferred other methods of adoption. Given that definitions of the adoption sources included in the best-worst experiment were not included, in an effort to avoid biasing the respondent, it is possible respondents may have considered different definitions of the adoption sources when making their choices. Surprisingly, adopting from a breeder, which might imply the dog was purposefully bred, was still preferred over adopting from a pet store in the RPL model. There are many negative reports and studies regarding pet stores such as insect infestations, internal parasites and congenital abnormalities in purchased puppies, which may negatively affect people’s preference for adopting from a pet store [43,44,45]. There are also reported differences in behavioral characteristics between dogs obtained as puppies from pet stores and those obtained from non-commercial breeders, including increased aggression, separation anxiety and house soiling [46]. Adopting from a municipal shelter was preferred over all other types of adoption in the RPL model. Although respondents with dogs were asked how they obtained their dog, the option adoption was not broken down further than adoption from a shelter or rescue. Future studies could go into more depth in regard to the type of shelter respondents obtained dogs from and compare those results to respondent preferences. Further research is needed to understand why people do not prefer adopting from pet stores. One possible explanation is that some people may be generally opposed to supporting pet stores that sell small pets due to concerns about the welfare conditions offered to animals within the store as well as prior to arriving there. Some may be concerned about ethical sourcing of pets sold in stores and generalize those across companion breeds available in retail stores. If other animals in the pet store, which were purposefully bred and are sold for a profit such as hamsters, are being advertised as “adoptable”, people may be unsure which animals are coming from shelters. Lusk et al. (2006), conjecture that if a consumer has imperfect information, the markets fail to provide a socially optimal allocation of resources, which would result in a decrease of utility [47]. If those looking to adopt a dog do not have complete, transparent information regarding the rearing of that animal, it is less likely they will be able to acquire a dog that was reared in a manner that meets their preferences. 

Younger respondents were more likely to be members of latent class 2, “adoption is great”, when compared to class 3, “breed matters”. Previous studies have shown that younger people may be more sensitive to dog welfare issues such as high euthanasia rates and high numbers of U.S. dogs in shelters and may have a greater concern for animal welfare [48]. Respondents with at least a college degree were less likely to be in class 2, “adoption is great”, when compared to their probability of being members of class 3, “breed matters”, Dogs have long been a status symbol from the lap dogs of the Victorian era to the expensive Russian Rottweiler guard dogs of the 90’s [49]. Ghirlanda et al. (2013) found that popularity spikes in different dog breeds were not correlated with breed characteristics but were more likely a function of fashion [50]. Breed rescues and “adopting” from a breeder may serve the dual purpose of ensuring a dog breed reflective of status, while maintaining general society’s preference for adoption as demonstrated by Bir et al. (2017).

Evidence of SDB is prevalent in self-reporting behaviors or perceptions related to many aspects of life. Widmar et al. (2016) found SDB in respondents’ reporting of holiday eating behaviors [32]. Additionally, Simons et al. (2015) found that non-active videogame playing young people underreported their sedentary gaming hours [51]. It is therefore unsurprising that differences were found between what respondents believed were important dog characteristics and what they believed others felt were important dog characteristics. Respondents indicated characteristics that result in a specific physical appearance such as breed and appearance were more important to others. It is true that the two characteristics breed and appearance are related and expectedly both showed very similar and high levels of social desirability bias (over 40%). Women and those who currently or previously had a dog showed a higher prevalence of SDB for appearance. It is possible dog owners may feel “guiltier” about finding the appearance of a dog important. Interestingly, Thorn et al. (2015), using a relationship quality survey, found that how “cute” a respondent found their dog positively affected the relationship quality between the respondent and their dog [52]. Given these findings, it is reasonable to question whether physical appearance of the dog may also matter in acquisition to some respondents, whether or not they are consciously aware or willing to admit it. The importance of appearance was also demonstrated by Reese et al. (2017) in a hedonic pricing model of dog and human characteristics [33]. They found people would pay less for a dog with a black coat when compared to a dog with a white or brown coat [33].

Conversely, less superficial characteristics such as compatibility with owner lifestyle, behavior and source of the dog were reportedly more important characteristics to the respondent, when compared to what they believed others found important. The importance of such characteristics can be identified by reasons why people relinquish their animals and factors that make an adoption successful. In a survey of shelter workers, Ellis et al. (2017) found that people relinquish their animals due to changes in lifestyle (new house, new partner), lack of planning for animal’s basic needs and aggression towards people in dogs, often due to poor training and socialization [53]. Using vignettes Hill and Murphy found that the two characteristics most likely to impact dog adoption success were behavior and size [54]. Dogs with at least some obedience training were more likely to be adopted successfully [54]. Younger respondents had a higher prevalence of SDB for physical health than older respondents. Others have found that people select dogs as a fad, or for social pressure reasons [49,50,52]. It is possible that younger respondents feel such pressure more than older respondents and if so, may attempt to hide a preference for fads by overstating their preference for what may be considered more socially responsible reasons for acquisition, such as health. As noted by Krishnan et al. (2014), some surveys suggest that younger, especially millennial consumers, are more concerned with corporate social responsibility than other age group segments and believe they have more influence on society as consumers than as voters [55]. It would therefore not be surprising if some younger respondents to the current survey replied in a manner consistent with advocating for what they might perceive to be ethical consumerism relative to dog acquisition, even if they themselves do not entirely adhere to such beliefs. However, Reese et al. (2007) found that whether the dog was microchipped, as well as the age of the dog impacted the price paid for a dog. This indicates that at least for some people, such characteristics often associated with safety (in terms of getting lost) and potentially the health of the animal are important [33]. Respondents with a lower education level exhibited higher SDB for genetic health, when compared to those with a higher education level. Future studies could ask dog owners more specific questions regarding the attributes that dog owners found appealing in their current dog, beyond the questions asked of dog owners in this study and use that as a factor in analyzing social desirability bias for dog characteristics. For example, if a respondent has paid for extensive obedience training for their own dog, they may have a higher preference for compatibility with owner lifestyle than other respondents. Additional factors such as experience with dog rescues, knowledge of the number of homeless dogs and ethnicity could all contribute to a better understanding of social desirability bias and its presence or lack thereof.

## 5. Conclusions

Dogs in general have a positive impact on the human condition. However, the first step in the human dog relationship is acquisition, which is often a contentious subject. Although people may hold strong personal preferences when it comes to dog acquisition, in general they prefer for choices to be available. In our previous findings (Bir et al., 2017), respondents overwhelmingly selected adoption as the most preferred method of dog acquisition. However, in context with their responses to other questions, including the observation that they also preferred to retain choices for dog procurement sources, questions were raised as to how people interpret the term, adoption, relative to dog ownership. Additionally, to what extent, if any, SDB may influence some people’s responses to inquiries about dog acquisition and its related ethical implications were considered in the current study.

The current study indicates that within the term adoption, which is often loosely used to describe acquiring a dog, people have preferences for the adoption source. Primarily, there is a preference for adopting from a municipal shelter, although when considering the latent class model, preferences vary across groups of people. The current study also investigated the extent to which SDB might influence people’s stated views on acquisition of dogs. The results suggested the likelihood that SDB influenced responses in several areas such as the importance of breed and appearance in acquiring a dog. Although SDB, when self-reporting dog characteristics of importance, may result in answers skewed more towards what is currently popular or socially acceptable, most respondents believed dogs could be bred ethically and responsibly. Acceptance of breeding as ethical and/or responsible is an important factor in sustainable pet ownership. Caution must be taken when considering responses about dog acquisition and preferred characteristics, as SDB may influence self-reported preferences and care must be taken to attempt to elicit stated preferences that may indeed be reflective of behaviors.

## Figures and Tables

**Table 1:** Respondent demographics in percent compared to the U.S. Census using a proportion test.

Demographic	Number of Respondents n = 767	Percentage of Survey Respondents n = 767	Percentage in U.S Census
*Gender*			
Female	414	54%	51%
*Age*			
18–24	77	10% ^†^	13% ^†^
25–34	134	17%	18%
35–44	138	18%	16%
45–54	148	19%	17%
55–65	133	17%	17%
65 and older	137	18%	19%
*Household Income*			
$0–$24,999	178	23%	22%
$25,000–$49,999	130	25%	23%
$50,000–$74,999	95	17%	17%
$75,000–$99,999	175	12%	12%
$100,000 and higher	189	23% ^†^	26% ^†^
*Education*			
Did not graduate from high school	25	3% ^†^	13% ^†^
Graduated from high school, did not attend college	207	27%	28%
Attended college, no degree earned	184	24% ^†^	21% ^†^
Attended college, associates or bachelor’s degree earned	246	32% ^†^	27% ^†^
Attended college, graduate or professional degree earned	105	14%	12%
*Region of Residence*			
Northeast	137	18%	18%
South	306	40% ^†^	21% ^†^
Midwest	153	20% ^†^	38% ^†^
West	171	22%	24%
*Has or has had a dog in the past 5 years*			
No	339	44%	^1^
Currently has a dog	348	45%	
Has had a dog in the past five years	142	19%	

^†^ The percentage of respondents and the U.S. census is statistically different at the 0.05 level. ^1^ Information regarding dog guardianship was not controlled to match the U.S census using quotas.

**Table 2:** Percentage of respondents who selected each method they would not use to obtain a dog. Multiple selections were permitted (n = 767) (number of selections = 2132).

Statement Regarding Dog Acquisition	Number of Respondents n = 767	Percentage of Respondents n = 767
*Are there any locations or methods you would NOT use to obtain a dog*		
Adoption (shelter or rescue organization)	79	10%
Breed them myself	241	31%
Purchased directly from a breeder (on site where dogs are bred/kept)	242	32%
Purchased directly from a breeder online (via the breeder’s website)	352	46%
Purchased from online retailer	425	55%
Purchased from pet store	324	42%
Stray	153	20%
Gift from friend/family member	90	12%
Other (i.e., parking lot)	226	29%

**Table 3:** Respondents’ beliefs regarding dog acquisition from a scale of 1 (agree) to 7 (disagree). Number of respondents, then percentage of respondents (number, percentage) (n = 767).

Statement	Agreement Level						
	1	2	3	4	5	6	7
The only responsible way to acquire a dog is through shelter/rescue	124, 16%	110, 14%	119, 16%	162, 21%	92, 12%	62, 8%	98, 13%
All dogs should be spayed/neutered	208, 27%	93, 12%	94, 12%	160, 21%	63, 8%	51, 7%	98, 13%
There is a dog overpopulation problem in the U.S.	225, 29%	103, 13%	86, 11%	169, 22%	61, 8%	52, 7%	71, 9%
Dogs in pet stores come from irresponsible breeders	119, 16%	99, 13%	96, 13%	228, 30%	93, 12%	53, 7%	79, 10%
Breeding of dogs for sale is socially irresponsible	121, 16%	79, 10%	97, 13%	203, 26%	97, 13%	71, 9%	99, 13%
People should be able to buy purebred dogs	259, 34%	105, 14%	94, 12%	134, 17%	65, 8%	48, 6%	62, 8%
People should have choices as to where/how to obtain dogs	245, 32%	143, 19%	99, 13%	120, 16%	67, 9%	40, 5%	53, 7%
Shelter dog populations would decrease if people stopped buying purebred dogs	158, 21%	87, 11%	77, 10%	199, 26%	77, 10%	75, 10%	94, 12%
Every shelter/rescue dog is adoptable	164, 21%	113, 15%	95, 12%	161, 21%	85, 11%	69, 9%	80, 10%
Importing of dogs for sale is irresponsible	241, 31%	105, 14%	81, 11%	163, 21%	63, 8%	57, 7%	57, 7%
Importing of dogs for adoption is irresponsible	188, 25%	95, 12%	101, 13%	164, 21%	71, 9%	72, 9%	76, 10%
The sale of dogs is socially irresponsible	104, 14%	66, 9%	83, 11%	191, 25%	109, 14%	96, 13%	118, 15%

**Table 4:** Percentage of respondents who have a dog or had a dog in the past 5 years who selected the response to specific dog ownership and care questions (n = 446).

Statement Regarding Dog Ownership or Care	Number of Dog Owners n = 446	Percentage of Dog Owners n = 446
*How have you acquired a dog ^1^*		
Adoption (shelter or rescue organization)	178	40%
Bred them myself	17	4%
Purchased directly from a breeder (on site where dogs are bred/kept)	90	20%
Purchased directly from a breeder online (via the breeder’s website)	25	6%
Purchased from online retailer	11	2%
Purchased from pet store	38	9%
Stray	59	13%
Gift from friend/family member	134	30%
Other (i.e., parking lot)	30	7%
*Reasons for acquiring dog in that manner ^1^*		
Impulse buy	44	10%
Reputation of the breeder	43	10%
Reputation of the rescue/shelter	66	15%
Previous experience	74	17%
Wanted a specific breed or type of dog	133	30%
Cost	52	12%
Guilt	12	3%
Peer Pressure	11	2%
It was the right thing to do	208	47%
Dog came with pet insurance	14	3%
Dog came with training/education	24	5%
Dog came with health guarantee	35	8%
*How often do/did you take your dog to the veterinarian*		
Never	24	5%
Once a year	210	47%
More than once a year	192	43%
I don’t know	20	4%
*How often do/did you use heart worm prevention on your dog*		
Continuously	244	55%
Sometimes	121	27%
Never	55	12%
I don’t know	26	6%
*How often do/did you use flea and tick prevention on your dog*		
Continuously	261	59%
Sometimes	134	30%
Never	37	8%
I don’t know	14	3%

^1^ Multiple selections were permitted.

**Table 5:** Cross tabulations between respondent characteristics and dog ownership and dog care.

	Demographic or Acquisition Preference	Have or Had a Dog in the Last 5 Years n = 446	Respondents Who Take Their Dog to the vet n = 402	Uses Heartworm Preventative n = 55	Uses Flea Preventative n = 37
Gender	Male n = 353	61% ^1^	49%	52% ^a2^	54%
	Female n = 414	56%	56%	44% ^b^	49%
Age	44 years old or less n = 349	66% ^a2^	57% ^a2^	53% ^a^	59% ^a2^
	45 years old or older n = 418	52% ^b^	49% ^b^	49% ^b^	45% ^b^
Income	Income $49,999 or less n = 367	53% ^a^	44% ^a^	40% ^a^	46% ^a^
	Income more than $50,000 n = 400	63% ^b^	60% ^b^	54% ^b^	57% ^b^
Education	No college degree n = 416	60%	53%	48%	54%
	At least a college degree n = 351	56%	52%	47%	48%
Region of Residence	Northeast n = 137	50% ^a^	46% ^a^	44% ^a^	46% ^a^
	South n = 306	66% ^b^	60% ^b^	55% ^b^	62% ^b^
	Midwest n = 153	51% ^a^	48% ^a^	44% ^a^	46% ^a^
	West n = 171	56% ^a^	49% ^a^	36% ^a^	42% ^a^
How respondent has acquired a dog	Has acquired a dog from a breeder n = 112	25% ^a^	93% ^a^	85% ^a^	88% ^a^
	Has not acquired a dog from a breeder n = 655	75% ^b^	46% ^b^	41% ^b^	45% ^b^
	Has acquired a dog through adoption n = 178	40% ^a^	97% ^a^	87% ^a^	90% ^a^
	Has not acquired a dog through adoption n = 589	60% ^b^	39% ^b^	36% ^b^	40% ^b^
	Has acquired a dog through purchase n = 140	31% ^a^	90.7% ^a^	84% ^a^	86% ^a^
	Has not acquired a dog through purchase n = 627	69% ^b^	44% ^b^	40% ^b^	44% ^b^
Acquisition Preference	Would not acquire a dog through adoption n = 79	52%	47%	46%	47%
	Would acquire a dog through adoption n = 688	59%	53%	48%	52%
	Would not acquire a dog through purchase n = 570	59%	55%^a^	49%	53%
	Would acquire a dog through purchase n = 197	55%	45%^b^	43%	47%

^1^ Percent is the percentage within the category. For example, 61% of men have or had a dog. ^2^ Within the table, matching letters (or lack of letters) indicate percentage of respondents for that category for example gender are not statistically different at the <0.001 level in regard to the percentage who responded yes to the statement, while differing letters indicate they are statistically different. For example, for the category gender and the column have or had a dog in the last 5 years, both male and female do not have a letter, indicating the percentages are not statistically different. However for the category age, the percentage of respondents who are 44 years old or less is marked with an a indicating it is statistically different than the percentage of respondents 45 years old or older.

**Table 6:** Multinomial logit model, random parameters logit model and latent class model results for most ethical way to adopt a dog, coefficients and preference shares (n = 767).

Ways to Adopt a Dog	Multinomial Logit Model	Random Parameters Logit Model			Latent Class Model					
					Coefficients (Standard Error)			Share of Preferences in Percentage		
	Coefficient (Standard Error)	Coefficient (Standard Error)	Standard Deviation (Standard Error)	Preference Share in Percentage	Class 1 “Municipal Animal Shelter First”	Class 2 “Adoption is Great!”	Class 3 “Breed Matters”	Class 1	Class 2	Class 3
Adopted from a pet store	−0.30 *** (0.03)	−0.64 *** (0.06)	2.02 *** (0.09)	3% ^a 1^	−0.60 ***^4^ (0.07)	0.28 ** (0.12)	−3.35 ** (1.42)	1%	22%	1%
Adopted from a municipal animal shelter	1.36 *** (0.03)	2.47 *** (0.08)	2.85 *** (0.10)	56% ^b^	3.95 *** (0.18)	0.30 ** (0.15)	−1.03 * (0.61)	66%	23%	14%
Adopted from a breed rescue	0.97 *** (0.30)	1.55 *** (0.06)	1.77 *** (0.07)	22% ^c^	2.80 *** (0.10)	0.13 ** (0.05)	−0.16 (0.42)	21%	19%	33%
Adopted from a privately-owned shelter	0.65 *** (0.03)	1.10 *** (0.05)	1.34 *** (0.06)	14% ^d^	2.12 *** (0.11)	0.10 (0.12)	−1.05 ** (0.50)	11%	19%	13%
Adopted from a breeder ^2^				5% ^e^				1%	17%	39%
Constant					1.89 ***	0.94 ***				
Less than 44 years old ^3^					0.61	1.45 ***				
At least a college Degree ^3^					−0.28	−0.89 *				
Class Probability					0.62	0.29	0.09			

^1^ Within the model, matching letters indicate the preference share is not statistically different, while differing letters indicate they are statistically different. For example, a preference share with an a is not statistically different within the model when compared to another preference share with an a. However a preference share with an a is statistically different within the model when compared to another preference share with a b. ^2^ To prevent multicollinearity, the category Adopted from a breeder was dropped from the model. All coefficients are in reference to this dropped variable. ^3^ Demographic constants were included in the latent class model as a predictor of class membership.^4^ * indicates statistical significance at the 0.10 level, ** at the 0.05 level, and *** at the <0.001 level.

**Table 7:** Comparison of characteristics respondents believe are most important when acquiring a dog and characteristics respondents think others believe are important when acquiring a dog on a scale from 1–4 with 1 be very important and 4 being very unimportant (n = 767).

Characteristic	Characteristics Respondents Look for or Believe are Most Important in Acquiring a Dog	Characteristics Respondents Believe Others Look for or Believe are Most Important in Acquiring a Dog
Breed	2.71 *** (1.04)	3.19 *** (0.94)
Appearance	2.81 *** (0.98)	3.30 *** (0.90)
Compatibility with owner lifestyle	3.38 *** (0.87)	3.21 *** (0.94)
Behavior	3.40 * (0.88)	3.32 * (0.90)
Genetic health	3.11 (0.92)	3.06 (0.94)
Physical health	3.34 (0.90)	3.34 (0.89)
Cost	2.92 (1.03)	2.99 (0.94)
Experience/reputation of source	3.13 (0.91)	3.10 (0.94)
Source of the dog	3.08 ** (0.97)	2.95 ** (0.10)

* Respondents vs. others statistically different at the ^1^ percent level. ** Respondents vs. others statistically different at the 0.05 percent level. *** Respondents vs. others statistically different at the 0.01 percent level.

**Table 8:** Cross tabulations of the presence of social desirability bias in choices relating to a specific dog characterization and respondent demographics. Percentage of respondents calculated as the percentage of each demographic category that exhibited social desirability bias (n = 767).

Category of Bias	Gender		Age		Income		Education		Dog in the Household	
	Male n = 353	Female n = 414	Less Than 44 Years Old n = 349	45 Years Old or Older n = 418	$49,999 or less n = 367	Over $50,000 n = 400	Less Than College n = 416	College or Higher n = 351	Has A Dog n = 446	Does Not Have a Dog n = 321
Breed bias n = 312	37.4	43.5	39.0	42.1	42.8	38.7	42.1	39.0	40.3	41.1
Appearance bias n = 312	36.5	44.0 ^a^	40.1	41.1	39.2	42.0	41.6	39.6	44.8 ^a^	34.9
Compatibility with owner lifestyle bias n = 183	21.5	25.8	25.8	22.2	23.4	24.3	21.9	26.2	24.7	22.7
Behavior bias n = 156	19.8	20.8	18.4	22.6	20.2	20.5	19.0	21.9	21.5	18.7
Genetic health bias n = 169	21.8	22.2	22.1	22.0	21.3	22.8	25.9 ^a^	18.8	21.7	22.4
Physical health bias n = 135	17.8	17.4	21.5 ^a^	14.4	17.7	17.5	14.7 ^a^	21.1	17.1	17.9
Cost bias n = 217	30.3	26.6	26.6	29.7	23.7 ^a1^	32.5	29.8	26.5	31.8 ^a^	23.4
Experience/reputation of source bias n = 196	26.3	24.6	22.9	27.8	24.3	26.8	25.2	25.9	23.3	28.7
Source of the dog bias n = 208	26.6	27.5	28.7	25.8	26.7	27.5	24.3	30.5	26.0	28.7

^1^ Within each category, matching letters, or no letters, indicate the percentage of respondents is not statistically different, while differing letters indicate they are statistically different at the 0.05 level. For example, within gender there are not letters next to male and female for breed bias which indicates the percentage of males is not statistically different than the percentage of females. Conversely, for appearance bias there is an a next to the percentage of females which indicates the percentage of males and females is statistically different for this category of bias.

## Supplementary Materials

The following are available online at http://www.mdpi.com/2076-2615/8/9/154/s1.
