# Supplementary material for: Exploring Social Desirability Bias in Perceptions of Dog Adoption: All’s Well that Ends Well? Or Does the Method of Adoption Matter?
Source: Animals (Basel). 2018 Sep 13;8(9):154. doi: 10.3390/ani8090154 (PMC6162534; doi:10.3390/ani8090154)
Supplement: Supplementary file 1 [file animals-08-00154-s001.pdf]

Supplementary File 1

Exploring Social Desirability Bias in Perceptions of Dog Adoption: All's Well that Ends Well? Or Does the Method of Adoption Matter? *Survey questions investigated in this study, including the demographics employed for quota purposes*

Q1.1 I am:

- ○ Male
- ○ Female

Q1.2 I am _____ years old.

- ○ Under 18
- ○ 18 - 24
- ○ 25 - 34
- ○ 35 - 44
- ○ 45 - 54
- ○ 55 - 65
- ○ 65 +

Q1.3 My household (including all other adults and children living in my household) has the following number of members (include yourself):

- ○ Adults (18 years and older) ____
- ○ Children (Under 18 years old) ____

Q1.4 My annual pre-tax, household income is:

- ○ $0-$24,999
- ○ $25,000-$49,999
- ○ $50,000-$74,999
- ○ $75,000-$99,999
- ○ $100,000 and higher

Q1.5 The best description of my educational background is:

- ○ Did not graduate from high school
- ○ Graduated from high school, Did not attend college
- ○ Attended College, No Degree earned
- ○ Attended College, Associates or Bachelor's Degree earned
- ○ Attended College, Graduate or Professional Degree earned

Q1.6 My region of residence is: __________. Select one option from the drop down menu.

- ○ Northeast (CT, ME, MA, NH, NJ, NY, PA, RI, VT) (1)
- ○ South (AL, AR, DE, DC, FL, GA, KY, LA, MD, MS, NC, OK, SC, TN, TX, VA, WV) (2)
- ○ Midwest (IL, IN, IA, KS, MI, MN, MO, NE, ND, OH, SD, WI) (3)
- ○ West (AK, AZ, CA, CO, HI, ID, MT, NV, NM, OR, UT, WA, WY) (4)

Q2.1 In the following exercise you will be asked to select the most ethical and the least ethical way to adopt a dog. It is important to note you will be asked **10 Identical Questions**, with **DIFFERENT** answer choices.
Q2.2 From the following set of sources, select the source that is the most ethical source to adopt a dog and the least ethical source to adopt a dog.

Most ethical		Least ethical
○	Adopted from a breeder	○
○	Adopted from a pet store	○
○	Adopted at a municipal animal shelter	○

Q2.3 From the following set of sources, select the source that is the most ethical source to adopt a dog and the least ethical source to adopt a dog.

Most ethical		Least ethical
○	Adopted from a privately owned shelter	○
○	Adopted from a breed rescue	○
○	Adopted from a breeder	○

Q2.4 From the following set of sources, select the source that is the most ethical source to adopt a dog and the least ethical source to adopt a dog.

Most ethical		Least ethical
○	Adopted at a municipal animal shelter	○
○	Adopted from a pet store	○
○	Adopted from a privately owned shelter	○

Q2.5 From the following set of sources, select the source that is the <u>most ethical</u> source to adopt a dog and the <u>least ethical</u> source to adopt a dog.

Most ethical		Least ethical
○	Adopted from a breed rescue	○
○	Adopted at a municipal animal shelter	○
○	Adopted from a pet store	○

Q2.6 From the following set of sources, select the source that is the most ethical source to adopt a dog and the least ethical source to adopt a dog.

Most ethical		Least ethical
○	Adopted from a breeder	○
○	Adopted at a municipal animal shelter	○
○	Adopted from a privately owned shelter	○

Q2.7 From the following set of sources, select the source that is the <u>most ethical</u> source to adopt a dog and the <u>least ethical</u> source to adopt a dog.

Most ethical		Least ethical
○	Adopted from a pet store	○
○	Adopted from a privately owned shelter	○
○	Adopted from a breed rescue	○

Q2.8 From the following set of sources, select the source that is the most ethical source to adopt a dog and the least ethical source to adopt a dog.

Most ethical		Least ethical
○	Adopted from a breed rescue	○
○	Adopted from a privately owned shelter	○
○	Adopted at a municipal animal shelter	○

Q2.9 From the following set of sources, select the source that is the most ethical source to adopt a dog and the least ethical source to adopt a dog.

Most ethical		Least ethical
○	Adopted at a municipal animal shelter (1)	○
○	Adopted from a breeder	○
○	Adopted from a breed rescue	○

Q2.10 From the following set of sources, select the source that is the most ethical source to adopt a dog and the least ethical source to adopt a dog.

Most ethical		Least ethical
○	Adopted from a privately owned shelter	○
○	Adopted from a breeder	○
○	Adopted from a pet store	○

Q2.11 From the following set of sources, select the source that is the most ethical source to adopt a dog and the least ethical source to adopt a dog.

Most ethical		Least ethical
○	Adopted from a pet store	○
○	Adopted from a breed rescue	○
○	Adopted from a breeder	○

Q3.1 Do you have a dog currently or have you had a dog in the past 5 years?

- ☐ No
- ☐ I currently have a dog
- ☐ I have had a dog in the past 5 years

Q3.2 How have you acquired a dog? (Select all that apply)

- ☐ Adoption (shelter or rescue organization)
- ☐ Bred them myself
- ☐ Purchased directly from a breeder (on-site where dogs are bred/kept)
- ☐ Purchased directly from a breeder online (via the breeder's website)
- ☐ Purchased from online retailer
- ☐ Purchased from pet store
- ☐ Stray
- ☐ Gift from friend/family member
- ☐ Other (i.e., parking lot)

Q3.3 For what reasons did you acquire your dog in that manner (Select all that apply)

- [ ] Impulse buy
- [ ] Reputation of the breeder
- [ ] Reputation of the rescue/shelter
- [ ] Previous experience
- [ ] Wanted a specific breed or type of dog
- [ ] Cost
- [ ] Guilt
- [ ] Peer pressure
- [ ] It was the right thing to do
- [ ] Dog came with pet insurance
- [ ] Dog came with training/education
- [ ] Dog came with health guarantee

Q3.4 How often do/did you take your dog to the veterinarian?

- ○ Never
- ○ Once a year
- ○ More than once a year
- ○ I don't know

Q3.5 How often do/did you use heart worm prevention on your dog?

- Continuously
- Sometimes
- Never
- I don't know

Q3.6 How often do/did you use flea and tick prevention on your dog?

- Continuously
- Sometimes
- Never
- I don't know

Q3.7 Are there any locations or methods you would **not** use to obtain a dog? (Select all that apply)

- [ ] Adoption (shelter or rescue organization)
- [ ] Bred them myself
- [ ] Purchased directly from a breeder (on-site where dogs are bred/kept)
- [ ] Purchased directly from a breeder online (via the breeder's website)
- [ ] Purchased from online retailer
- [ ] Purchased from pet store
- [ ] Stray
- [ ] Gift from friend/family member
- [ ] Other (i.e., parking lot)

Q3.8 What characteristics do <u>you</u> look for or believe are most important in acquiring a dog

	1 (Very unimportant)	2	3	4 (Very important)
Breed	○	○	○	○
Appearance	○	○	○	○
Compatibility with owner lifestyle	○	○	○	○
Behavior	○	○	○	○
Genetic health	○	○	○	○
Physical health	○	○	○	○
Cost	○	○	○	○
Experience/reputation of source	○	○	○	○
Source of the dog	○	○	○	○

Q3.9 What are characteristics you believe other people look for or find most important when acquiring a dog?

	1 (Very unimportant)	2	3	4 (Very important)
Breed	○	○	○	○
Appearance	○	○	○	○
Compatibility with owner lifestyle	○	○	○	○
Behavior	○	○	○	○
Genetic Health	○	○	○	○
Physical Health	○	○	○	○
Experience/reputation of source	○	○	○	○
Cost	○	○	○	○
Source of the dog	○	○	○	○

Q3.10 I believe that dogs can be bred responsibly.

- ○ Yes
- ○ No

Q3.11 I believe that dogs can be bred ethically.

- ○ Yes
- ○ No

Q3.12 Please rate your level of agreement or disagreement with the following statements on a scale of:

	1 (Completely agree)	2	3	4	5	6	7 (Completely disagree)
The only responsible way to acquire a dog is through shelter/rescue.	○	○	○	○	○	○	○
All dogs should be spayed/neutered.	○	○	○	○	○	○	○
There is a dog overpopulation problem in the US.	○	○	○	○	○	○	○
Dogs in pet stores come from irresponsible breeders.	○	○	○	○	○	○	○
Breeding of dogs for sale is socially irresponsible.	○	○	○	○	○	○	○
People should be able to buy purebred dogs.	○	○	○	○	○	○	○
People should have choices as to where/how to obtain dogs.	○	○	○	○	○	○	○
Shelter dog populations would decrease if people stopped buying purebred dogs.	○	○	○	○	○	○	○
Every shelter/rescue dog is adoptable.	○	○	○	○	○	○	○

Importing of dogs for sale is irresponsible.	○	○	○	○	○	○	○
Importing of dogs for adoption is irresponsible.	○	○	○	○	○	○	○
The sale of dogs is socially irresponsible.	○	○	○	○	○	○	○

Q4.1 Thank you for your time in completing this survey. Your input will strengthen our research and help us obtain more accurate conclusions. If you wish to add any comments please feel free to do so here:
